# Non-infarcted myocardium bears the weight in CVD

**DOI:** 10.18632/aging.101890

**Published:** 2019-03-25

**Authors:** Hui Zhou, Hafisyatul Zainal, Valentina O. Puntmann

**Affiliations:** 1 Institute of Experimental and Translational Cardiac Imaging, DZHK Centre for Cardiovascular Imaging, Frankfurt, Germany; 2 Department of Radiology, XiangYa Hospital, Central South University, Changsha, Hunan, China; 3 Department of Cardiology, Universiti Teknologi MARA (UiTM), Sg. Buloh, Malaysia

**Keywords:** Cardiovascular Magnetic Resonance (CMR), myocardial injury, Late Gadolinium Enhancement (LGE), ejection fraction (EF), native T1

Cardiovascular disease (CVD) remains the leading cause of cardiac morbidity and mortality in the entire world population. Heart failure (HF) is the fastest growing cardiac diagnosis, with an annual incidence of 10 cases per 1000 people in individuals older than 65 [1]. This is partly a reflection of an aging population and success of treatment of acute coronary syndromes with reduced premature mortality due to ischaemic heart disease (IHD), as well as increasing ability to recognise non-ischaemic - intrinsic myocardial processes- due to advances in genetics and imaging. The conventional imaging predictors of outcome in CVD patients primarily include left ventricular ejection fraction (LVEF) and late gadolinium enhancement (LGE) using cardiovascular magnetic resonance (CMR). LVEF represents the main universal, as well as the multimodality biomarker of risk stratification. It is also used to guide therapy; when accompanied by symptoms of failing heart, LVEF ≤35% is the default clinical index that triggers the cascade of therapeutic measures. Whereas LVEF is an indirect measure of myocardial damage, inferred by the consequent functional impairment, LGE by CMR can directly visualize myocardial injury. LGE can also distinguish between the irreversible, transmural (100% wall thickness) myocardial injury and the one, where less myocardial layers have been affected (<50% wall thickness) and where the segments’ function could improve with revascularization, thus salvaging the hibernating myocardium [2]. Prognostically, LGE exceeds the prediction of all-cause and cardiac mortality [3,4] provided by LVEF; the amount of scar, also known as the LGE extent, has been shown to be prognostically informative in a number of cardiac conditions including, IHD and non-ischaemic cardiomyopathies [5,6]. Yet, despite the excellent evidence, LGE is neither integrated into the major risk nor treatment models, partially on the grounds that CMR, as a method and the expertise, may not be sufficiently available.

It is increasingly recognized that the focus on functional impairment or the myocardial damage provides a short view of what matters in achieving either longevity or reprieve from the devasting CVD. This insight is partially afforded through increasingly scar-free patient population, which is either revascularized early, or sufficiently protected from the vulnerable plaque rupture by two decades of statin therapies. And yet, aging population still faces an epidemic of HF. As we increasingly investigate population of CVD patients in receipt of modern immediate revascularization therapy, we find that the infarct sizes are relatively small and LVEF frequently preserved. By an overall reduced importance of the two traditional parameters, LVEF and LGE, the focus is shifting onto the non-infarcted myocardium. This message reflects our recently reported findings of a prospective, observational, multicenter longitudinal study, incorporating the two classical imaging markers, LVEF and LGE, as well as the novel quantitative tissue characterization measures of non-infarcted myocardium, based on T1 mapping with CMR, the native T1 and ECV. We found that native T1, the gadolinium contrast free measure, of non-infarcted myocardium was an independent predictor of survival and major cardiocerebrovascular events (MACCE) [6]. Moreover, with increasing native T1 values, the likelihood of events was were significantly higher; the group of patients with values in upper tertile had 6.2-times greater likelihood of poor survival and 4.5-times for MACCE, compared to those with native T1 within normal range. Notably, the presence of LGE remained an important predictor of outcome, however only in the subgroup of patients with considerably large scars, and thus, the functional impairment.

This evidence is important for several reasons. Firstly, it is a testament to the importance of a direct measure of myocardial pathology. Native T1 reflects the presence and severity of myocardial changes and pathological myocardial remodeling in non-infarcted myocardium, directly relating to the intrinsic myocardial disease, such as the presence of myocardial oedema, inflammation, diffuse fibrosis and infiltration [7,8]. In the presence of CAD, the non-infarcted myocardium is affected by intrinsic disease mechanisms, which are pathophysiologically different and separate from the ischaemic - vascular myocardial injury as a result of myocardial infarction [6]. In addition to patients with known CAD, T1 mapping indices have also been shown to relate to prognosis in a number of other cardiac conditions, including non-ischaemic dilated cardiomyopathy, diabetes, and amyloidosis [5,6]. Secondly, native T1 is a quantitative biomarker; thus unsurprisingly, the prognosis is proportionally related to the disease severity. Thirdly, T1 mapping is a sensitive measure pathological myocardial remodeling and inflammation, and may be useful in selecting patients most likely to benefit from its therapeutic modulation. It is thus possible that native T1 measures reflects a modifiable substrate, rendering its reduction of native T1 a potential therapeutic target providing means of risk modification and improved prognosis. Sustained monitoring of native T1 levels may allow for an individual optimization of treatment, possibly ahead of the symptom manifestation and development of phenotypically expressed, often an irreversible disease. Thus, CMR with T1 mapping provides an important refinement of the current concept of risk assessment and may help to overcome an important gap in clinical management and discovery of therapies.
